# Isolation of a Novel Polyketide from *Neodidymelliopsis* sp.

**DOI:** 10.3390/molecules26113235

**Published:** 2021-05-27

**Authors:** Melissa M. Cadelis, Hugo Gordon, Alex Grey, Soeren Geese, Daniel R. Mulholland, Bevan S. Weir, Brent R. Copp, Siouxsie Wiles

**Affiliations:** 1 School of Chemical Sciences, University of Auckland, Private Bag 92019, Auckland 1142, New Zealand; hgor595@aucklanduni.ac.nz (H.G.); b.copp@auckland.ac.nz (B.R.C.); 2 Bioluminescent Superbugs Lab, School of Medical Sciences, University of Auckland, Private Bag 92019, Auckland 1142, New Zealand; alex.grey@auckland.ac.nz (A.G.); s.geese@auckland.ac.nz (S.G.); d.mulholland@auckland.ac.nz (D.R.M.); 3 Manaaki Whenua – Landcare Research, Private Bag 92170, Auckland 1142, New Zealand; WeirB@landcareresearch.co.nz

**Keywords:** fungi, polyketide, natural product

## Abstract

Fungi have become an invaluable source of bioactive natural products, with more than 5 million species of fungi spanning the globe. Fractionation of crude extract of *Neodidymelliopsis* sp., led to the isolation of a novel polyketide, (2*Z*)-cillifuranone (**1**) and five previously reported natural products, (2*E*)-cillifuranone (**2**), taiwapyrone (**3**), xylariolide D (**4**), pachybasin (**5**), and *N*-(5-hydroxypentyl)acetamide (**6**). It was discovered that (2*Z*)-cillifuranone (**1**) was particularly sensitive to ambient temperature and light resulting in isomerisation to (2*E*)-cillifuranone (**2**). Structure elucidation of all the natural products were conducted by NMR spectroscopic techniques. The antimicrobial activity of **2**, **3**, and **5** were evaluated against a variety of bacterial and fungal pathogens. A sodium [1-^13^C] acetate labelling study was conducted on *Neodidymelliopsis* sp. and confirmed that pachybasin is biosynthesised through the acetate polyketide pathway.

## 1. Introduction

**Figure 1:**
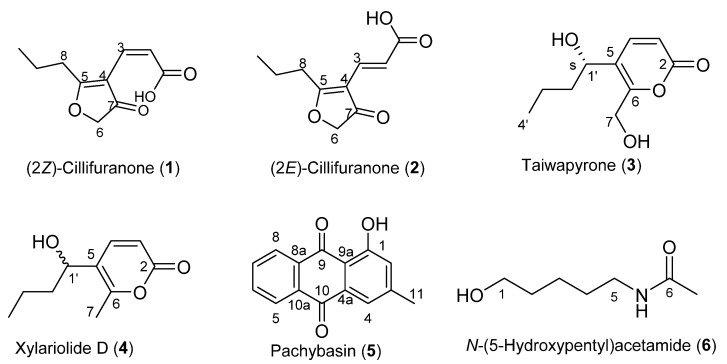
Structures of isolated natural products **1**–**6**.

Fungi are well known producers of antimicrobial natural products and have given society some of the largest medical breakthroughs in our time. More than 5 million species of fungi span the globe providing an abundance of potential natural products that can be screened for bioactivity [1]. We have an ongoing interest in identifying novel natural products from fungal isolates from the International Collection of Microorganisms from Plants (ICMP) [2]. Using whole cell assays, we found *Neodidymelliopsis* sp. (ICMP 11463) to show antibacterial activity against *Staphylococcus aureus* and *Mycobacterium abscessus* but not *Escherichia coli* or *Pseudomonas aeruginosa*. Fractionation of the crude extract of *Neodidymelliopsis* sp. led to the isolation of two furanones, (2*Z*)-cillifuranone (**1**) and (2*E*)-cillifuranone (**2**); two α-pyrones, taiwapyrone (**3**) and xylariolide D (**4**); one anthraquinone, pachybasin (**5**); and one acetamide, *N*-(5-hydroxypentyl)acetamide (**6**) (Figure 1). Of the six natural products isolated, (2*Z*)-cillifuranone (**1**) was novel, while compounds **2**–**6** have been previously reported [3,4,5,6,7]. Upon further investigation, (2*Z*)-cillifuranone (**1**) was found to be unstable, spontaneously isomerising to (2*E*)-cillifuranone (**2**) in ambient temperature and light.

**Figure 2:**
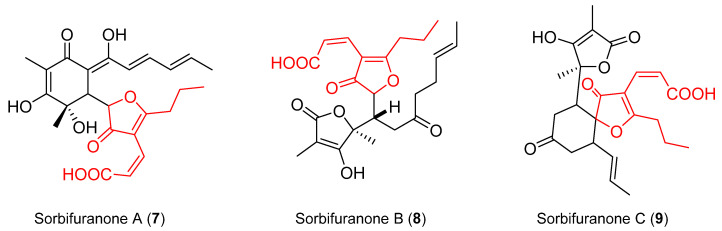
Sorbifuranone A (**7**), sorbifuranone B (**8**), sorbifuranone C (**9**), with (2*Z*)-cillifuranone fragments highlighted in red.

Previous reports have shown that (2*E*)-cillifuranone (**2**) was isolated from the same fungi that afforded sorbifuranone A–C (**7**–**9**), which have (2*Z*)-cillifuranone (**1**) incorporated in their structure (Figure 2) [8]. It was postulated that (2*Z*)-cillifuranone (**1**) was an anticipated natural product but was the unfavoured isomer of the two [4,8]. Herein, we report the isolation and structure elucidation of (2*Z*)-cillifuranone (**1**), along with known natural products **2**–**6**.

## 2. Results and Discussion

### 2.1. Isolation and Structure Elucidation

**Figure 3:**
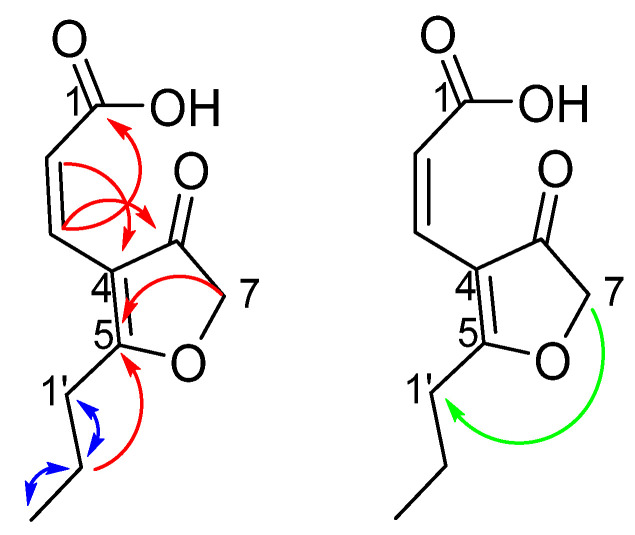
Selected HMBC (red) and COSY correlations (blue) and the long-range ^5^*J*_HH_ COSY correlation (green) for (2*Z*)-cillifuranone (**1**).

Freeze-dried plates (dry weight 108.6 g) inoculated with *Neodidymelliopsis* sp. (ICMP 11463) were extracted with a combination of MeOH and CH_2_Cl_2_. Preliminary fractionation of the brown gum was carried out using C_8_ reversed-phase flash chromatography, eluting with a MeOH/H_2_O gradient, to generate five fractions (F1–F5). Further purification of F3 and F4 by a combination of Sephadex LH20 and silica gel column chromatography afforded compounds **1**–**6** (Figure 3).

High resolution ESI mass spectrum of compound **1**, isolated as pale-yellow needles, showed a sodiated adduct of *m*/*z* 219.0638 [M + Na^+^] which established a molecular formula of C_10_H_12_O_4_. The ^1^H-NMR spectrum of **1** exhibited one set of alkene protons (δ_H_ 6.49 (d, *J* = 11.6 Hz, H-3) and 6.01 (d, *J* = 11.6 Hz, H-2)), three methylenes (δ_H_ 4.62 (s, H_2_-7), 2.60 (t, *J* = 7.0 Hz, H_2_-1**′**) and 1.75–1.70 (m, H_2_-2**′**)), and one methyl group (δ_H_ 0.99 (t, *J* = 7.0 Hz, H_3_-3**′**)) (Table 1). The ^13^C-NMR spectrum identified 10 signals including three carbonyl carbons (δ_C_ 201.2 (C-8), 193.4 (C-5), and 169.5 (C-1)), three olefinic carbons (δ_C_ 114.9 (C-4), 124.4 (C-2), 130.0 (C-3)), three methylenes (δ_C_ 76.1 (C-7), 32.9 (C-1**′**), and 20.3 (C-2**′**)), and, finally, one methyl carbon (δ_C_ 14.0 (C-3**′**)). Analysis of COSY NMR data established the presence of two distinct proton spin-systems; two protons (δ_H_ 6.49, δ_H_ 6.01) were attributed to a *Z*-alkene as they had a *J*_HH_ coupling constant of 11.6 Hz, while a second spin-system was attributed to an alkyl chain (δ_H_ 2.60, δ_H_ 1.75–1.70, and δ_H_ 0.99) (Figure 3). HMBC correlations observed between δ_H_ 6.49 (H-3) and δ_C_ 201.2 (C-8) and 169.5 (C-1) combined with correlations from δ_H_ 6.01 (H-2) to δ_C_ 114.9 (C-4) established the position of a carboxylic acid, two carbonyl carbons and the attachment point of the *Z*-alkene at position C-4. In addition, HMBC correlations between δ_H_ 1.75–1.70 (H_2_-2**′**) and 193.4 (C-5) established the position of the alkyl chain at C-5. Issues arose with placement of the remaining methylene carbon at C-7. COSY correlations between δ_H_ 4.62 (H_2_-7) and δ_H_ 2.60 (H_2_-1**′**) suggested the remaining methylene must be a near H_2_-1**′**; however, no HMBC correlations to the alkyl chain were observed. An HMBC correlation from δ_H_ 4.62 to δ_C_ 193.4 suggested a furanone ring. Thus, compound **1** was determined to be (2*Z*)-cillifuranone. The spectroscopic data showed many similarities to those observed for (2*E*)-cillifuranone with differences centred on the alkene, where compound **1** contains a *Z*-alkene (δ_H_ 6.49, δ_C_ 130.0) and (δ_H_ 6.01, δ_C_ 124.4) and **2** contains an *E*-alkene (δ_H_ 7.32, δ_C_ 132.6) and (δ_H_ 6.83, δ_C_ 118.7). The most notable similarity with the present study was an unusual ^5^*J*_HH_ COSY correlation observed between H_2_-7 and H_2_-1**′** which was also reported by Weise et al. during the structure elucidation of **2** [4].

Efforts to purify (2*Z*)-cillifuranone (**1**) generally ended with an *E*/*Z* mixture. Issues in separation occurred due to the negligible polarity between the two isomers. Several methods to achieve separation based on polarity differences were attempted, including low-pressure silica gel, Diol-bonded silica gel, and C_8_ column chromatography; however, none of these methods proved effective. Of the separation methods attempted, silica gel column chromatography using a CH_2_Cl_2_:MeOH gradient solvent system proved the most successful leading to isolation of 0.9 mg of (2*Z*)-cillifuranone (**1**). Unfortunately, isomerisation had begun before full characterisation could be completed.

**Figure 4:**
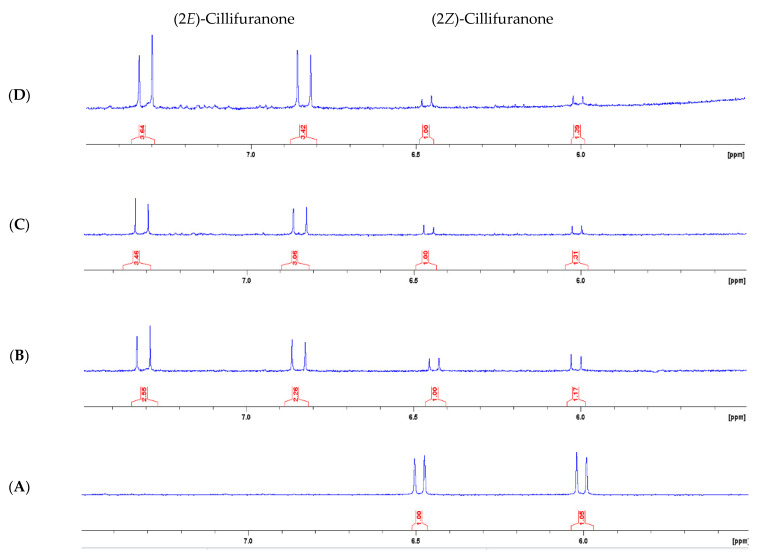
Time-controlled experiment of (2*Z*)-cillifuranone (**1**) isomerisation. (**A**): time zero, (**B**): three months after time zero, (**C**): six months after time zero, (**D**): nine months after time zero.

An ^1^H-NMR time course experiment was used to explore the ease of conversion of (2*Z*)-cillifuranone (**1**) to (2*E*)-cillifuranone (**2**), and to answer the possibility that conversion of **1** to **2** occurred during chromatography and with time. The time course experiment involved dissolving **1** in *d*_4_-methanol and storing the sample open to light at room temperature and acquiring a ^1^H-NMR spectrum every three months (Figure 4). Over a nine-month period, **1** was observed to isomerise to **2**. After three months, approximately 70% of (2*Z*)-cillifuranone (**1**) had isomerised, while, after nine months, approximately 77% had isomerised.

This time-controlled experiment clearly demonstrated that (2*Z*)-cillifuranone (**1**) is ambient light and temperature sensitive, isomerising to (2*E*)-cillifuranone (**2**). Since **2** appeared to be the more stable isomer, this could explain some of difficulties that we had in acquiring **1** in amounts required for characterisation. The extraction procedure could have led to **1** isomerising to **2**. The standard extraction procedure used does not seek to minimise light exposure and the process of drying initial fractions collected from reversed-phase C_8_ column chromatography requires removal of water that is achieved under reduced pressure in a 40 °C water bath. Further extractions of *Neodidymelliopsis* sp. were undertaken in the absence of light and with water baths at room temperature. These changes were made to minimise isomerisation of **1**. However, no further isolation of pure (2*Z*)-cillifuranone (**1**) was achieved.

The structures of the other four known compounds were identified as taiwapyrone (**3**), xylariolide D (**4**), pachybasin (**5**), and *N*-(5-hydroxypentyl)acetamide (**6**) by comparison of NMR data with literature [5,9,10].

### 2.2. Evaluation of Bioactivity

**Figure 5:**
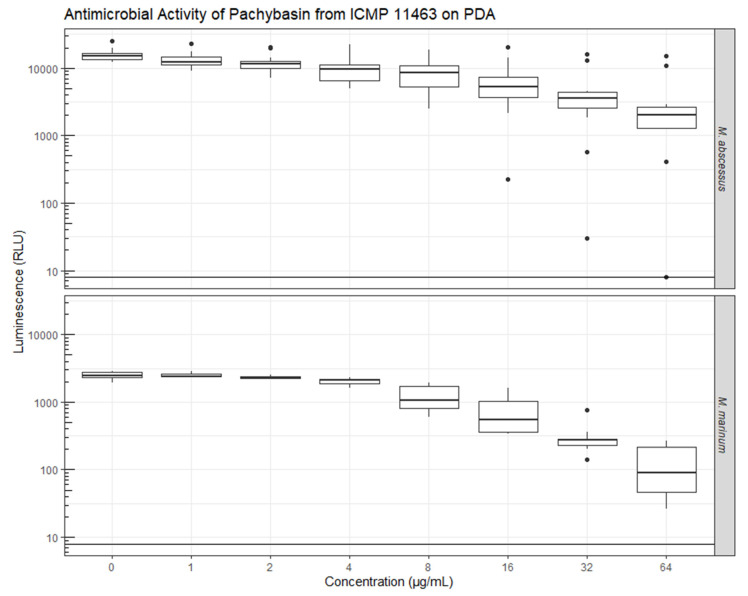
Antimicrobial activity of **5** against *M. abscessus* and *M. marinum.* The raw data (Supplementary Materials II) has been deposited online on Figshare.com (doi:10.17608/k6.auckland.14503584).

During initial inhouse whole cell screening, *Neodidymelliopsis* sp. (ICMP 11463) showed antibacterial activity against *Staphylococcus aureus* and *Mycobacterium abscessus* but not *Escherichia coli* or *Pseudomonas aeruginosa*. Three of the six compounds isolated from *Neodidymelliopsis* sp. were evaluated for their bioactivity against *Mycobacterium abscessus* and *M. marinum* using inhouse assays, and against a more extensive panel of microorganisms (*Escherichia coli*, *Klebsiella pneumonia*, *Pseudomonas aeruginosa*, *Acinetobacter baumannii*, Methicillin-resistant *Staphylococcus aureus*, *Candida albicans*, and *Cryptococcus neoformans*) by the Community for Open Antimicrobial Drug Discovery (COADD) at The University of Queensland. COADD test compound activity as inhibition of microbial growth at a single concentration of 32 µg/mL and their assays did not identify any significant inhibition of growth for any compound tested (Table 2). Similarly, we found no activity for **2** and **3** against *M. abscessus* or *M. marinum* (data not shown). However, we did find some anti-mycobacterial activity for **5** with Minimum Inhibitory Concentrations (MIC) of 32 and 64 µg/mL for *M. marinum* and *M. abscessus*, respectively (Figure 5).

### 2.3. Proposed Biosynthesis

**Figure 6:**
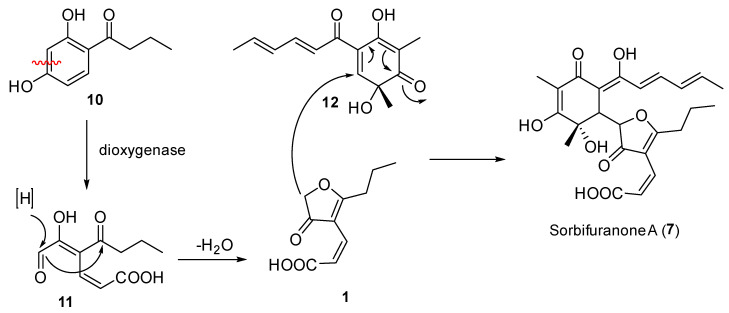
Proposed biosynthesis of sorbifuranone A (**7**) using (2*Z*)-cillifuranone (**1**) by Bringmann et al. [8].

In 2010, three secondary metabolites, sorbifuranone A–C (**7**–**9**), were isolated from *Penicillium chryogenum* [8]. These metabolites contained a (2*Z*)-cillifuranone fragment incorporated into the larger sorbifuranone structures (Figure 2). The authors postulated a biosynthesis for sorbifuranone A (**7**) using (2*Z*)-cillifuranone (**1**) and sorbicillinol (**12**) as intermediates (Figure 6) [8]. Recent studies on the chemistry of *Penicillium chryogenum* led to the discovery of (2*E*)-cillifuranone (**2**) that was found in conjunction with sorbifuranone A–C (**7**–**9**) [4]. An unconfirmed signal in their UV-spectrum led the researchers to postulate that (2*Z*)-cillifuranone (**1**) must exist and be the unfavoured isomer in the fungus [4].

**Figure 7:**
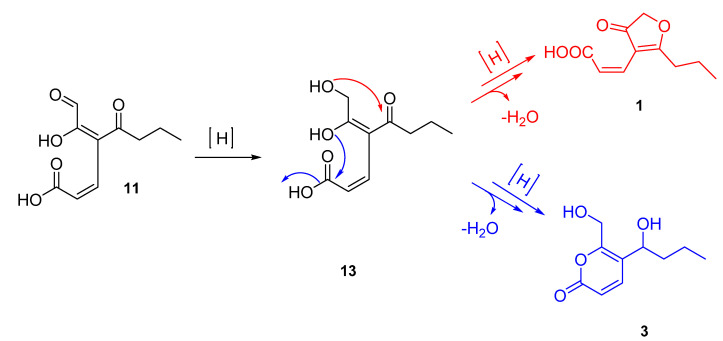
Proposed biosynthesis of (2*Z*)-cillifuranone (**1**) (red) and taiwapyrone (**3**) (blue).

We speculate that it is possible to achieve the biosynthesis of both (2*Z*)-cillifuranone and taiwapyrone using the previously proposed intermediate **11** as a common biosynthetic intermediate (Figure 7). The biosynthesis of **1** can begin with putative enzymatic oxidation of **10** to form **11**, which can be mediated by dioxygenase followed by reduction of the aldehyde to a secondary hydroxyl to form **13**. From **13**, it is possible for two different cyclization pathways to lead to two different products. The first cyclization (red) begins with attack of the ketone by the allylic alcohol to form the furanone ring followed by expulsion of water, the resulting product is (2*Z*)-cillifuranone (**1**). The second possible cyclization (blue) pathway involves the vinyl alcohol attacking the carbonyl of the acid forming the six membered α-pyrone ring with the expulsion of water. Subsequent reduction of the ketone forms taiwapyrone (**3**).

Having such commonly related intermediates allows *Neodidymelliopsis* sp. to have flexibility in production of these two secondary metabolites. Depending on the conditions *Neodidymelliopsis* sp. is exposed to, the fungus can respond with rapid production of (2*Z*)-cillifuranone (**1**) or taiwapyrone (**3**). Given that some investigations of the chemistry of *Neodidymelliopsis* sp. yielded no (2*E*/*Z*)-cillifuranone, it is plausible that the pathway is sensitive to growing conditions.

### 2.4. Sodium [1-^13^C] Acetate Incorporation Study

Due to the lack of biosynthetic knowledge for compounds, such as taiwapyrone and (2*E*)-cillifuranone, a sodium [1-^13^C] acetate labelling study was conducted to test the hypothesis that these compounds were produced through the acetate pathway. The organism was regrown in solid culture with 1 mg/mL sodium [1-^13^C] acetate incorporated in the agar. Standard extraction and fractionation unfortunately only yielded pachybasin (**5**), with no chromatographic evidence observed for **1**–**4** and **6**.

**Figure 8:**
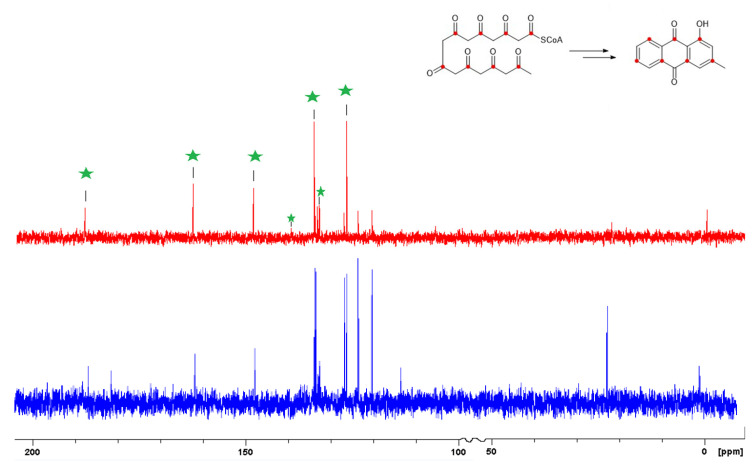
^13^C-NMR spectra of unlabelled (blue) and biosynthetically labelled (red) pachybasin. Green stars: ^13^C enhanced carbon signals.

The ^13^C-NMR spectra of labelled (Figure S12, Supplementary Materials) and unlabelled pachybasin (**5**) (Figure 8 and Figure S12) were overlapped and the peak heights for C-11 were matched to identify any enhanced carbon signals in the labelled spectrum. A number of peaks were observed with enhanced intensities of 7.4*(C-3), 6.4*(C-9), 6.2*(C-8), 6.1*(C-6), 6.1*(C-4a), 5.5*(C-1), 5.0*(C-10a) (*relative to C-11) (Table 3). The acetate that was used during this experiment was enriched at the carbonyl which allowed a predicted labelling pattern based on a related anthraquinone, chrysophanol, the biosynthesis of which has been extensively studied [11]. The predicted labelling pattern agreed with the labelling pattern observed for pachybasin (**5**) (Figure 8), providing conclusive evidence that pachybasin is synthesised through the acetate polyketide pathway.

Further examination of the enriched carbon chemical shifts and determination of pairs of chemical shifts led to the reassignment of three quaternary carbons (C-3, C-4a, and C-9a) previously assigned incorrectly in isolation reports of pachybasin (**5**) (Table 3).

**Figure 9:**
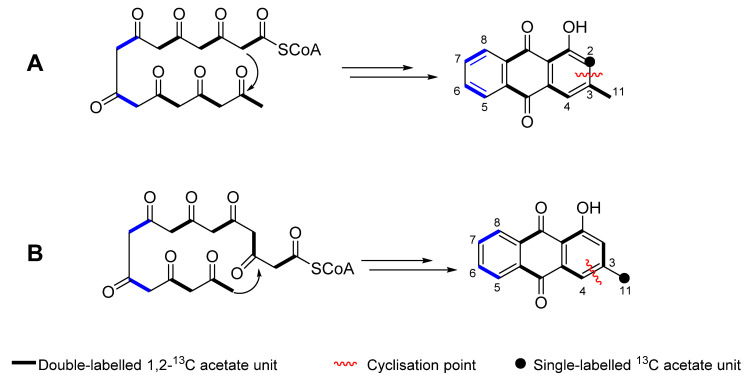
Predicted F**′**-mode (**A**) and F**′**-mode (**B**) octaketide folding for pachybasin (**5**).

To determine the exact polyketide folding mode that leads to pachybasin, a second experiment would need to be devised using 1,2-^13^C sodium acetate. In the established F folding mode, the octaketide cyclization point lies between C-2 and C-3 [11,13]. The use of double labelled acetate would identify intact acetate units, with F-mode leaving C-2 as a single labelled position (Figure 9). In contrast, the proposed F**′**-mode folding leaves C-11 as a single labelled position [11,13]. Both cyclization modes preserve the F-mode labelling pattern. To generate the evidence required to prove the F**′** folding mode, a ^13^C-COSY experiment would be acquired, with the ^13^C-^13^C connectivity being used to definitively establish the cyclization mode that leads to pachybasin.

## 3. Materials and Methods

### 3.1. General Experimental Procedures

Infrared spectra were recorded on a Perkin-Elmer spectrometer 100 Fourier Transform infrared spectrometer (PerkinElmer, Boston, MA, USA) equipped with a universal ATR accessory. HRMS data were acquired on a Bruker micrOTOF QII spectrometer (Bruker Daltonics, Bremen, Germany). NMR spectra were recorded on a Bruker Avance DRX-400 spectrometer (Bruker, Karlsruhe, Germany) or an Avance III-HD 500 spectrometer (Bruker, Karlsruhe, Germany) operating at 400 or 500 MHz for ^1^H nuclei and 100 or 125 MHz for ^13^C nuclei. Proto-deutero solvent signals were used as internal references (CD_3_OD: δ_H_ 3.31, δ_C_ 49.00 and CDCl_3_: δ_H_ 7.26, δ_C_ 77.16). For ^1^H-NMR, the data are quoted as position (δ), relative integral, multiplicity (s = singlet, d = doublet, t = triplet, q = quartet, m = multiplet, br = broad), coupling constant (*J*, Hz) and assignment to the atom. The ^13^C-NMR data are quoted as position (δ) and assignment to the atom. Flash column chromatography was carried out using either Diol bonded silica (40–63 µm) (Luknova, Mansfield, MA, USA), Davisil silica gel (40–63 µm) (Merck, Munich, Germany), or C_8_ reversed-phase (40–63 µm) solid support (Luknova, Mansfield, MA, USA). Gel filtration flash chromatography was carried out on Sephadex LH-20 (Merck, Munich, Germany). Thin layer chromatography was conducted on Merck (Munich, Germany) DC-plastikfolien Kieselgel 60 F254 plates. All solvents used were of analytical grade or better and/or purified according to standard procedures.

### 3.2. Fungal Material

Fungal material was provided by Manaaki Whenua—Landcare Research, a New Zealand Crown Research Institute (Auckland, New Zealand) responsible for the curation of the International Collection of Microorganisms from Plants (ICMP). The ascomycete fungus *Neodidymelliopsis* was described as a new genus in 2015 as a group of plant-associated *Phoma*-like fungi [14]. Culture ICMP 11463 was isolated in October 1991 from a leaf spot on New Zealand native *Pittosporum*. As the ITS sequence does not closely match any known *Neodidymelliopsis* species, it may be a novel species in this genus [14]. Freezer stocks were made by growing the fungus on 1.5% potato dextrose agar (PDA) plate and excising small cubes of agar (5–6 mm in length) from the fungus’ growing edge. These cubes were placed within a cryovial containing 1 mL of 10% glycerol. The cryovials were rested for 1 h, after which the remaining liquid glycerol was removed, and the tubes were stored at −80 °C.

### 3.3. Fermentation, Extraction and Isolation

Fifty-five PDA plates were inoculated with ICMP 11463 and incubated at room temperature for 4 weeks. Fully grown fungal plates were freeze-dried (108.6 g, dry weight) and extracted with MeOH (2 × 500 mL) for 4 h followed CH_2_Cl_2_ (2 × 500 mL) overnight. Combined organic extracts were concentrated under reduced pressure to afford a red/brown gum (0.51 g). The crude product was subjected to C_8_ reversed-phase column chromatography eluting with a gradient of H_2_O/MeOH to afford five fractions (F1–F5). F3 was subjected to purification by Sephadex LH-20, eluting with MeOH, to afford eight fractions (A1–A8). Further purification of fraction A4 by silica gel column chromatography, eluting with gradient CH_2_Cl_2_/MeOH, afforded taiwapyrone (**3**) (24 mg) and *N*-(5-hydroxypentyl)acetamide (**6**) (7 mg). Purification of F4 by Sephadex LH-20, eluting with MeOH, generated three fractions (B1–B3) of which B2 afforded pachybasin (**5**) (16 mg). Additional purification of B1 by silica gel column chromatography, eluting with gradient *n*-hexane/EtOAc, generated four fractions (C1–C4) of which C2 contained (2*E*)-cillifuranone (**2**) (30 mg). Fraction C3 afforded xylariolide D (**4**) (2 mg), (2*Z*)-cillifuranone (**1**) (0.9 mg) and a 0.4:1 mixture of **3** and **1** (4 mg) after further purification by silica gel column chromatography, eluting with gradient CH_2_Cl_2_/MeOH.

#### 3.3.1. (2*Z*)-Cillifuranone (**1**)

Pale yellow needles; R_f_ (CH_2_Cl_2_:MeOH, 9:1) 0.40; m.p. 145–147 °C; IR (ATR) v_max_ 3340, 2471, 2243, 2216, 2128, 2071, 1385, 1121, 973, 827 cm^−1^; ^1^H-NMR (400 MHz, CD_3_OD) δ 6.49 (d, *J* = 11.6 Hz, H-3), 6.01 (d, *J* = 11.6 Hz, H-2), 4.62 (2H, s, H_2_-7), 2.60 (2H, t, *J* = 7.0 Hz, H_2_-1**′**), 1.75–1.70 (2H, m, H_2_-2**′**), 0.99 (3H, t, *J* = 7.0 Hz, H_3_-3**′**); ^13^C-NMR (100 MHz, CD_3_OD) δ 201.2 (C-8), 193.4 (C-5), 169.5 (C-1), 130.0 (C-3), 124.4 (C-2), 114.9 (C-4), 76.1 (C-7), 32.9 (C-1’), 20.3 (C-2’), 14.0 (C-3’); (+)−HRESIMS *m*/*z* 219.0635 [M + Na]^+^ (calcd C_10_H_12_O_4_Na, 219.0633).

#### 3.3.2. (2*E*)-Cillifuranone (**2**)

Yellow oil; R_f_ (*n*-hexane:EtOAc, 9:1) 0.17; IR (ATR) v_max_ 3055, 1704, 1625, 1571, 1421, 1265, 1186, 982, 896, 734, 704 cm^−1^; ^1^H-NMR (400 MHz, CD_3_OD) δ 7.32 (1H, d, *J* = 15.6 Hz, H-3), 6.83 (1H, d, *J* = 15.6 Hz, H-2), 4.67 (2H, s, H_2_-7), 2.76 (2H, t, *J* = 7.4 Hz, H_2_-1**′**), 1.81–1.72 (2H, m, H_2_-2’), 1.02 (3H, t, *J* = 7.4 Hz, H_3_-3’); ^13^C-NMR (100 MHz, CD_3_OD) δ 201.9 (C-8), 196.7 (C-5), 170.9 (C-1), 132.6 (C-3), 118.7 (C-2), 112.6 (C-4), 76.3 (C-7), 31.6 (C-1’), 21.1 (C-2’), 14.0 (C-3’) (^1^H- and ^13^C-NMR data agreed with literature) [9]; (+)−HRESIMS *m*/*z* 219.0638 [M + Na]^+^ (calcd C_10_H_12_O_4_Na, 219.0633).

#### 3.3.3. Taiwapyrone (**3**)

Yellow oil; R_f_ (CH_2_Cl_2_:MeOH, 9:1) 0.23; IR (ATR) v_max_ 3333, 2946, 2835, 2164, 1648, 1450, 1412, 1111, 1019 cm^−1^; [α]_D_^21^ = −44.8 (*c* = 0.35, MeOH) ([α]_D_^20^ = −48.5 (*c* = 0.33, MeOH) literature) [3]; ^1^H-NMR (400 MHz, CD_3_OD) δ 7.68 (1H, d, *J* = 9.6 Hz, H-4), 6.34 (1H, d, *J* = 9.6 Hz, H-3), 4.76–4.72 (1H, m, H-1’), 4.43 (2H, s, H_2_-7), 1.78–1.69 (1H, m, H-2’_A_), 1.60–1.51 (1H, m, H-2’_B_), 1.49–1.39 (1H, m, H-3’_A_), 1.35–1.28 (1H, m, H-3’_B_), 0.95 (3H, t, *J* = 7.2 Hz, H_3_-4’); ^13^C-NMR (100 MHz, CD_3_OD) δ 164.2 (C-2), 159.9 (C-6), 145.8 (C-4), 121.8 (C-5), 116.3 (C-3), 67.9 (C-1’), 59.0 (C-7), 40.5 (C-2’), 19.9 (C-3’), 14.2 (C-4’) (^1^H- and ^13^C-NMR data agreed with literature) [5]; (+)−HRESIMS *m*/*z* 221.0791 [M + Na]^+^ (calcd C_10_H_14_NaO_4_, 221.0784).

#### 3.3.4. Xylariolide D (**4**)

Colourless oil; R_f_ (CH_2_Cl_2_:MeOH, 9:1) 0.43; ^1^H-NMR (400 MHz, CDCl_3_) δ 7.51 (1H, d, *J* = 9.4 Hz, H-4), 6.23 (1H, d, *J* = 9.4 Hz, H-3), 4.65 (1H, t, *J* = 6.7 Hz, H-1’), 2.27 (3H, s, H_3_-7), 1.80–1.72 (1H, m, H-2’_A_), 1.52–1.42 (1H, m, H-2’_B_), 1.40–1.35 (1H, m, H-3’_A_), 1.29–1.24 (1H, m, H-3’_B_), 0.95 (3H, t, *J* = 7.6 Hz, H_3_-4’) (^1^H-NMR data agreed with literature) [5]; ^1^H-NMR (400 MHz, CD_3_OD) δ 7.62 (1H, d, *J* = 9.6 Hz, H-4), 6.23 (1H, d, *J* = 9.6 Hz, H-3), 4.61 (1H, t, *J* = 6.9 Hz, H-1’), 2.28 (3H, s, H_3_-7), 1.77–1.68 (1H, m, H-2’_A_), 1.58–1.47 (1H, m, H-2’_B_), 1.46–1.35 (1H, m, H-3’_A_), 1.34–1.23 (1H, m, H-3’_B_), 0.95 (3H, t, *J* = 7.0 Hz, H_3_-4’); ^13^C-NMR (100 MHz, CD_3_OD) δ 164.6 (C-2), 159.6 (C-6), 146.0 (C-4), 121.8 (C-5), 114.1 (C-3), 68.5 (C-1’), 40.3 (C-2’), 19.8 (C-3’), 17.0 (C-7), 14.1 (C-4’); (+)−HRESIMS *m*/*z* 205.0829 [M + Na]^+^ (calcd C_10_H_14_O_3_Na, 205.0835).

#### 3.3.5. Pachybasin (**5**)

Yellow solid; R_f_ (*n*-hexane:EtOAc, 1:1) 0.87; IR (ATR) v_max_ 1672, 1637, 1591, 1361, 1276, 1223, 757, 710 cm^−1^; ^1^H-NMR (400 MHz, CDCl_3_) δ 12.58 (1H, s, OH), 8.32–8.29 (2H, m, H-5, H-8), 7.82–7.79 (2H, m, H-6, H-7), 7.67 (1H, d, *J* = 1.2 Hz, H-4), 7.12–7.10 (1H, m, H-2), 2.47 (3H, s, H_3_-11); ^13^C-NMR (100 MHz, CDCl_3_) δ 188.3 (C-9), 182.9 (C-10), 162.9 (C-1), 148.8 (C-3), 134.6 (C-6), 134.3 (C-7), 133.8 (C-10a), 133.5 (C-8a), 133.3 (C-4a), 127.5 (C-5), 127.0 (C-8), 124.3 (C-2), 121.0 (C-4), 114.3 (C-9a), 22.4 (C-11) (^1^H-NMR data agreed with literature) [10]; (+)−HRESIMS *m*/*z* 261.0529 [M + Na]^+^ (calcd C_15_H_10_O_3_Na, 261.0528).

#### 3.3.6. *N*-(5-Hydroxypentyl)acetamide (**6**)

Clear oil; R_f_ (CH_2_Cl_2_:MeOH, 1:1) 0.46; IR (ATR) v_max_ 3286, 3097, 3062, 2937, 2864, 1633, 1559, 1437, 1370, 1266, 1042, 1005, 896, 733, 702 cm^−1^; ^1^H-NMR (400 MHz, CDCl_3_) δ 3.64 (2H, t, *J* = 6.3 Hz, H_2_-1), 3.25 (2H, q, *J* = 7.0 Hz, H_2_-5), 1.97 (3H, s, H_3_-7), 1.62–1.50 (4H, m, H_2_-2, H_2_-4), 1.45–1.39 (2H, m, H_2_-3); ^13^C-NMR (100 MHz, CDCl_3_) δ 170.4 (C-6), 62.6 (C-1), 39.6 (C-5), 32.3 (C-2), 29.4 (C-4), 23.4 (C-3), 23.2 (C-7); (+)−HRESIMS *m*/*z* 168.0989 [M + Na]^+^ (calcd C_7_H_15_NO_2_Na, 168.0995).

### 3.4. Sodium [1-^13^C] Acetate Incroporated Acetate Fermentation of Neodidymelliopsis sp. ICMP 11463

#### 3.4.1. Fungal Material

Agar plates with a concentration of 1 mg/mL (sodium [1-^13^C] acetate/PDA agar) were prepared. The sodium [1-^13^C] acetate agar plates were inoculated with 1 cm^3^ cubes of pre-prepared *Neodidymelliopsis* sp. ICMP 11463 PDA plates. Plates were sealed with parafilm, inverted, and incubated at 37 °C for 18 h.

#### 3.4.2. Extraction and Isolation

Seventy-four PDA plates inoculated with ICMP 11463 were freeze-dried (39.61 g, dry weight) and extracted with MeOH (2 × 500 mL) and CH_2_Cl_2_ (1 × 500 mL). The combined organic extracts were concentrated under reduced pressure to afford a red/brown gum (5.01 g). Preliminary fractionation of the crude extract was carried out using reversed-phase C_8_ column chromatography, eluting with MeOH/H_2_O to generate five fractions (F1–F5). Purification of F3, F4, and F5 using a Sephadex LH-20, eluting with MeOH, followed by diol-bonded silica gel column chromatography, eluting with gradient *n*-hexane/EtOAc, afforded pachybasin (**5**) (20 mg) as a yellow solid.

### 3.5. Antimicrobial Assays of Pure Compounds

Antimicrobial evaluation against *M. abscessus* and *M. marinum* was undertaken using inhouse assays. *M. abscessus* BSG301 and *M. marinum* BSG101 [15] are stable bioluminescent derivatives transformed with the integrating plasmid pMV306G13ABCDE [16]. This allows light production to be used as a surrogate for bacterial viability [15,17,18,19]. Mycobacterial cultures were grown with shaking (200 rpm) in Middlebrook 7H9 broth (Fort Richard, Auckland, New Zealand) supplemented with 10% Middlebrook ADC enrichment media (Fort Richard, Auckland, New Zealand), 0.4% glycerol (Sigma-Aldrich, St. Louis, MO, USA), and 0.05% tyloxapol (Sigma-Aldrich, St. Louis, MO, USA). *M. abscessus* was grown at 37 °C and *M. marinum* at 28 °C.

Cultures were grown until they reached stationary phase (approximately 3–5 days for *M. abscessus* BSG301 and 7–10 days for *M. marinum* BSG101) and then diluted in Mueller Hinton broth II (MHB) (Fort Richard, Auckland, NewZealand) supplemented with 10% Middlebrook ADC enrichment media and 0.05% tyloxapol to give an optical density at 600 nm (OD_600_) of 0.001 which is the equivalent of ~10^6^ bacteria per mL. Pure compounds were dissolved in DMSO and added in duplicate to the wells of a black 96-well plate (Nunc, Thermo Scientific, Waltham, MA, USA) at doubling dilutions with a maximum concentration of 128 μg/mL. Then, 50 μL of diluted bacterial culture was added to each well of the compound containing plates giving final compound concentrations of 0–64 μg/mL and a cell density of ~5 × 10^5^ CFU/mL. Rifampicin (Sigma-Aldrich, St. Louis, MO, USA) was used as positive control at 1000 μg/mL for *M. abscessus* and 10 μg/mL for *M. marinum*. Between measurements, plates were covered, placed in a plastic box lined with damp paper towels, and incubated with shaking at 100 rpm at 37 °C for *M. abscessus* and 28 °C for *M. marinum*. Bacterial luminescence was measured at regular intervals using a Victor X-3 luminescence plate reader (PerkinElmer, Boston, MA, USA) with an integration time of 1 s. We have defined the MIC as causing a 1 log reduction in light production, as previously described [17,18]. More detailed protocols are available at protocols.io (accessed on 24 May 2021) [20,21].

Antimicrobial evaluation against *S. aureus* ATCC 43300 (MRSA), *E. coli* ATCC 25922, *P. aeruginosa* ATCC 27853, *Klebsiella pneumoniae* ATCC 700603, *Acinetobacter baumannii* ATCC 19606, *Candida albicans* ATCC 90028, and *Cryptococcus neoformans* ATCC 208821 was undertaken at the Community for Open Antimicrobial Drug Discovery at The University of Queensland, Australia, according to their standard protocol [22]. The tested strains were cultured in either Luria broth (LB) (In Vitro Technologies, USB75852, Victoria, Australia), nutrient broth (NB) (Becton Dickson, 234000, New South Wales, Australia), or MHB at 37 °C overnight. A sample of culture was then diluted 40-fold in fresh MHB and incubated at 37 °C for 1.5−2 h. The compounds were added in duplicate to the wells of a 96-well plate (Corning 3641, nonbinding surface) at a concentration of 64 μg/mL. The resultant mid log phase cultures were diluted to the final concentration of 1 × 10^6^ CFU/mL; then, 50 μL were added to each well of the compound containing plates giving a final compound concentration of 32 μg/mL and a cell density of 5 × 10^5^ CFU/mL. All plates were then covered and incubated at 37 °C for 18 h. Resazurin was added at 0.001% final concentration to each well and incubated for 2 h before MICs were read by eye.

For the antifungal assay, fungi strains were cultured for 3 days on YPD agar at 30 °C. A yeast suspension of 1 × 10^6^ to 5 × 10^6^ CFU/mL was prepared from five colonies. These stock suspensions were diluted with yeast nitrogen base (YNB) (Becton Dickinson, 233520, New South Wales, Australia) broth to a final concentration of 2.5 × 10^3^ CFU/mL. The compounds were added in duplicate to the wells of a 96-well plate (Corning 3641, nonbinding surface) at a concentration of 64 μg/mL and a final volume of 50 μL. Then, 50 μL of the fungi suspension that was previously prepared in YNB broth to the final concentration of 2.5 × 10^3^ CFU/mL were added to each well of the compound-containing plates, giving a final compound concentration of 32 μg/mL. Plates were covered and incubated at 35 °C for 36 h without shaking. *C. albicans* MICs were determined by measuring the absorbance at OD_530_. For *C. neoformans*, resazurin was added at 0.006% final concentration to each well and incubated for a further 3 h before MICs were determined by measuring the absorbance at OD_570−600_.

Colistin and vancomycin were used as positive bacterial inhibitor standards for Gram-negative and Gram-positive bacteria, respectively. Fluconazole was used as a positive fungal inhibitor standard for *C. albicans* and *C. neoformans*. The antibiotics were provided in 4 concentrations, with 2 above and 2 below its MIC value, and plated into the first 8 wells of Column 23 of the 384-well NBS plates. The quality control (QC) of the assays was determined by the antimicrobial controls and the Z’-factor (using positive and negative controls). Each plate was deemed to fulfil the quality criteria (pass QC), if the Z’-factor was above 0.4, and the antimicrobial standards showed full range of activity, with full growth inhibition at their highest concentration, and no growth inhibition at their lowest concentration.

## 4. Conclusions

A study of *Neodidymelliopsis* sp. led to the isolation of a novel polyketide, (2*Z*)-cillifuranone (**1**), and five known natural products: (2*E*)-cillifuranone (**2**), taiwapyrone (**3**), xylariolide D (**4**), pachybasin (**5**), and *N*-(5-hydroxypentyl)acetamide (**6**). Three of the six natural products were tested against a range of bacterial and fungal pathogens; however, none of the natural products showed any significant antimicrobial activity, with the exception of **5,** which showed some activity against *M. abscessus* and *M. marinum* with MIC of 64 and 32 µg/mL, respectively. A sodium [1-^13^C] acetate incorporation experiment was performed to determine the biosynthesises of these natural products. Only pachybasin (**5**) was isolated from the labelled extract of *Neodidymelliopsis* sp., with the pattern of label incorporation being consistent with biosynthesis via cyclization of an acetate polyketide pathway-derived octaketide.

## Figures and Tables

**Table 1:** ^1^H and ^13^C chemical shift (CD_3_OD) for (2*Z*)-cillifuranone (**1**).

Position	δ_H_ (m, *J* in Hz) ^a^	δ_C_^b^
1	–	169.5
2	6.01 (d, 11.6)	124.4
3	6.49 (d, 11.6)	130.0
4	–	114.9
5	–	193.4
7	4.62 (s)	76.1
8	–	201.2
1′	2.60 (t, 7.0)	32.9
2′	1.75–1.70 (m)	20.3
3′	0.99 (t, 7.0)	14.0

^a^ Data recorded at 400 MHz; ^b^ Data recorded at 100 MHz.

**Table 2:** Antimicrobial activities of **2**, **3**, and **5**.

Compound	Percentage Growth Inhibition ^a^						
	*S. a. ^b^*	*P. a. ^c^*	*E. c. ^d^*	*K. p. ^e^*	*A. b. ^f^*	*C. a. ^g^*	*C. n. ^h^*
**2**	12.28	4.76	−0.69	8.93	1.4	0.03	−5.11
**3**	5.32	15.56	15.94	19.17	13.89	8.13	−2.28
**5**	53.02	5.27	3.32	11.9	22.71	5.75	−13.06

All values presented as the mean (*n* = 2). ^a^ Percentage inhibition at a single dose test of 32 µg/mL; ^b^ *Staphylococcus aureus* ATCC 43300 (MRSA); ^c^ *Pseudomonas aeruginosa* ATCC 27853; ^d^ *Escherichia coli* ATCC 25922; ^e^ *Klebsiella pneumoniae* ATCC 700603; ^f^ *Acinetobacter baumannii* ATCC 19606; ^g^ *Candida albicans* ATCC 90028; ^h^ *Cryptococcus neoformans* ATCC 208821.

**Table 3:** Assignment of ^13^C-NMR resonances using labelled pachybasin (**5**) in comparison to literature.

Position	δ_C_	Relative Intensity ^a^	δ_C_^b^
1	162.9	5.5	162.7
2	124.3	1.2	124.1
3	148.8	7.4	114.0
4	121.0	1.2	120.7
4a	133.3	6.1	148.6
5	127.5	1.3	127.3
6	134.6	6.1	134.4
7	134.3	1.2	134.0
8	127.0	6.2	126.1
8a	133.5	1.0	133.5
9	188.3	6.4	187.9
9a	114.3	1.0	133.1
10	182.9	1.0	182.6
10a	133.8	5.0	133.2
11	22.4	1.0	22.2

**^a^** relative intensity to C-11; ^b^ data from Liu et al. [12].

## Data Availability

The data presented in this study are available in the supplementary material and online at Figshare.com (doi:10.17608/k6.auckland.14503584, accessed on 27 May 2021).

## Supplementary Materials

The following are available online: the ^1^H-, ^13^C-, COSY, HSQC, HMBC, and NOESY NMR spectra for compound **1**, ^1^H- and ^13^C-NMR spectra of **4** and **6**, ^13^C-NMR spectra of **5**. The raw data for the antimicrobial activity of **5** against *M. abscessus* and *M. marinum* are also available online.

## Sample Availability

Samples of the compounds **2**–**6** are available from the authors.
