# Supplementary material for: Isolation of a Novel Polyketide from Neodidymelliopsis sp
Source: Molecules. 2021 May 27;26(11):3235. doi: 10.3390/molecules26113235 (PMC8199022; doi:10.3390/molecules26113235)
Supplement: Supplementary file 1 [file molecules-26-03235-s001.zip › molecules-1227304-supplementary/molecules-1227304-supplementary for conversion pdf/Supplementary Material file 1.pdf]

molecules

Supplementary Material

Isolation of a Novel Polyketide from *Neodidymelliopsis* sp.

Melissa M. Cadelis [1,2,*], Hugo Gordon [1], Alex Grey [2], Soeren Geese [2], Daniel R. Mulholland [2], Bevan S. Weir [3], Brent R. Copp [1,†] and Siouxsie Wiles [2,*,†]

1 School of Chemical Sciences, University of Auckland, Private Bag 92019, Auckland 1142, New Zealand; hgor595@aucklanduni.ac.nz (H.G.); b.copp@auckland.ac.nz (B.R.C.)
2 Bioluminescent Superbugs Lab, School of Medical Sciences, University of Auckland, Private Bag 92019, Auckland 1142, New Zealand; alex.grey@auckland.ac.nz (A.G.); s.geese@auckland.ac.nz (S.G.); d.mulholland@auckland.ac.nz (D.R.M.)
3 Manaaki Whenua – Landcare Research, Private Bag 92170, Auckland 1142, Auckland, New Zealand; WeirB@landcareresearch.co.nz
* Correspondence: m.cadelis@auckland.ac.nz (M.M.C.); s.wiles@auckland.ac.nz (S.W.) Tel.: (M.M.C.); (S.W.)
† These authors contributed equally to this work.

Contents

Figure S1 ^{1}H-NMR spectrum (400 MHz, CD_3OD) of **1** **S2**

Figure S2 ^{1}H-NMR spectrum (400 MHz, CD_3OD) of **1** and **3** **S3**

Figure S3 ^{13}C-NMR spectrum (100 MHz, CD_3OD) of **1** and **3** **S4**

Figure S4 COSY spectrum (CD_3OD) of **1** and **3** **S5**

Figure S5 HSQC spectrum (CD_3OD) of **1** and **3** **S6**

Figure S6 HMBC spectrum (CD_3OD) of **1** and **3** **S7**

Figure S7 ^{1}H-NMR spectrum (400 MHz, CD_3OD) of **4** **S8**

Figure S8 ^{13}C-NMR spectrum (100 MHz, CD_3OD) of **4** **S9**

Figure S9 ^{1}H-NMR spectrum (400 MHz, $CDCl_3$) of **6** **S10**

Figure S10 ^{13}C-NMR spectrum (100 MHz, $CDCl_3$) of **6** **S11**

Figure S11 ^{13}C-NMR spectrum (100 MHz, $CDCl_3$) of **5** **S12**

Figure S12 ^{13}C-NMR spectrum (100 MHz, $CDCl_3$) of 1-^{13}C acetate labelled **5** **S13**

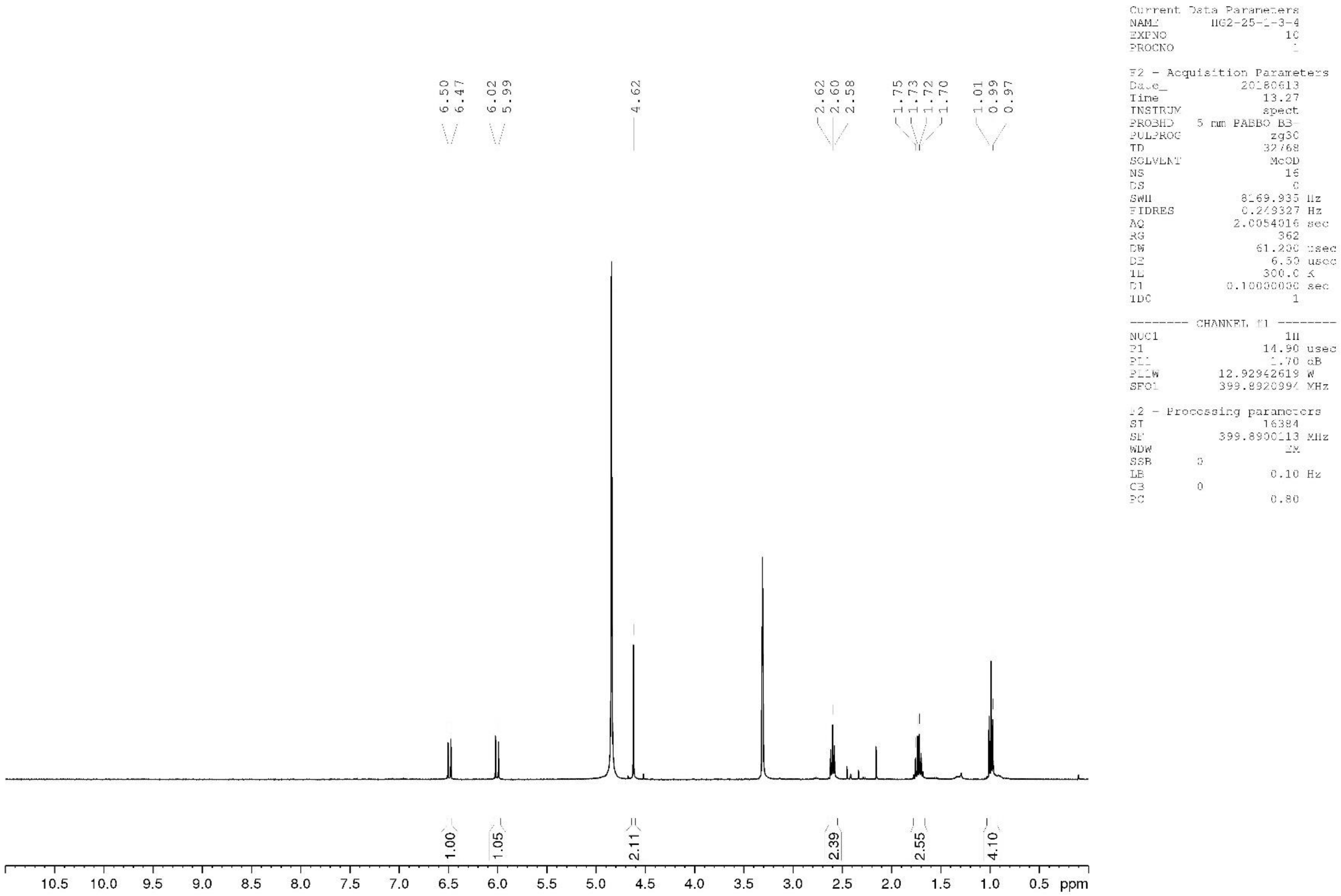

Figure S1 ^{1}H-NMR spectrum (400 MHz, CD_3OD) of **1**

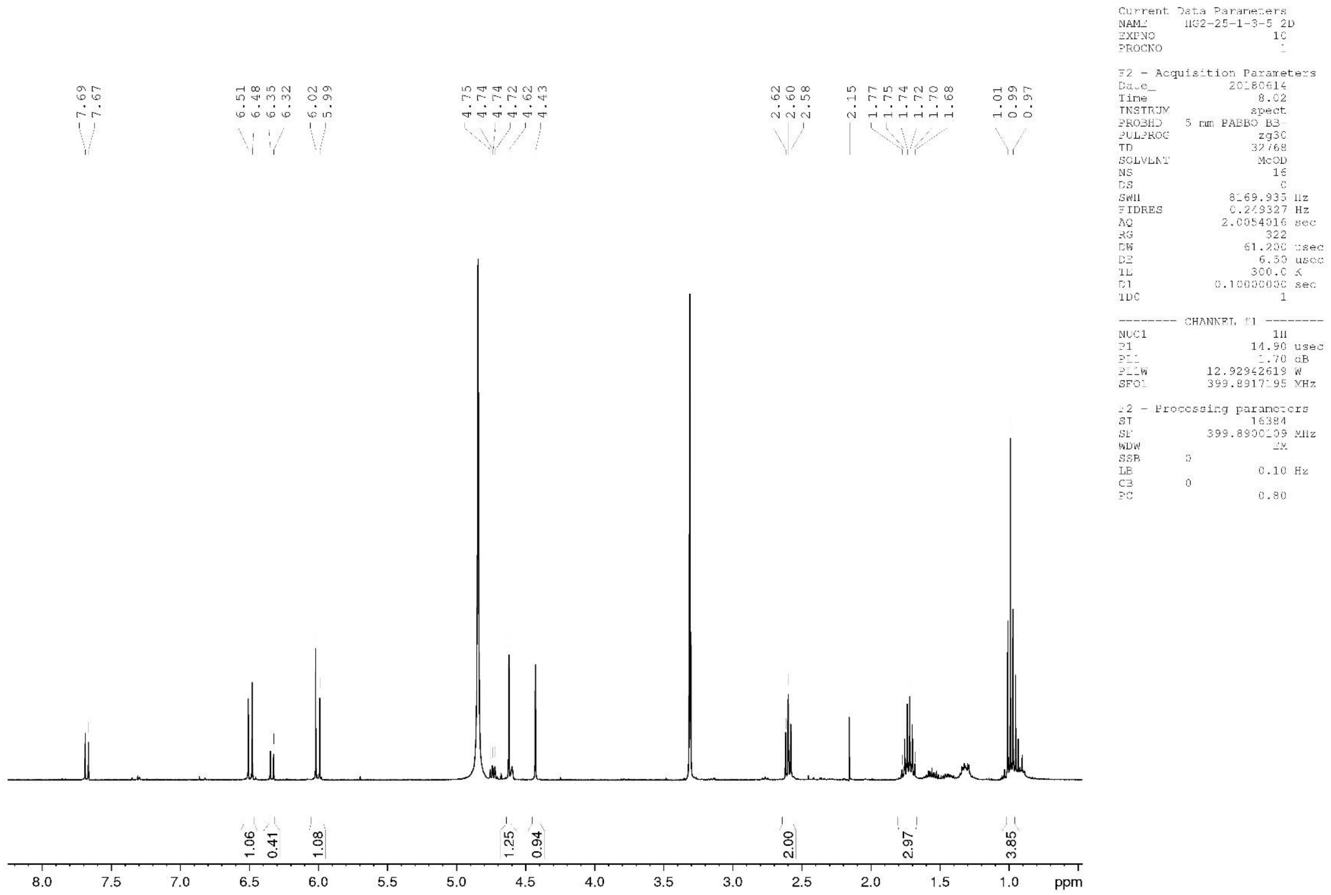

Figure S2 ^{1}H-NMR spectrum (400 MHz, CD_3OD) of a mixture of **1** and **3**

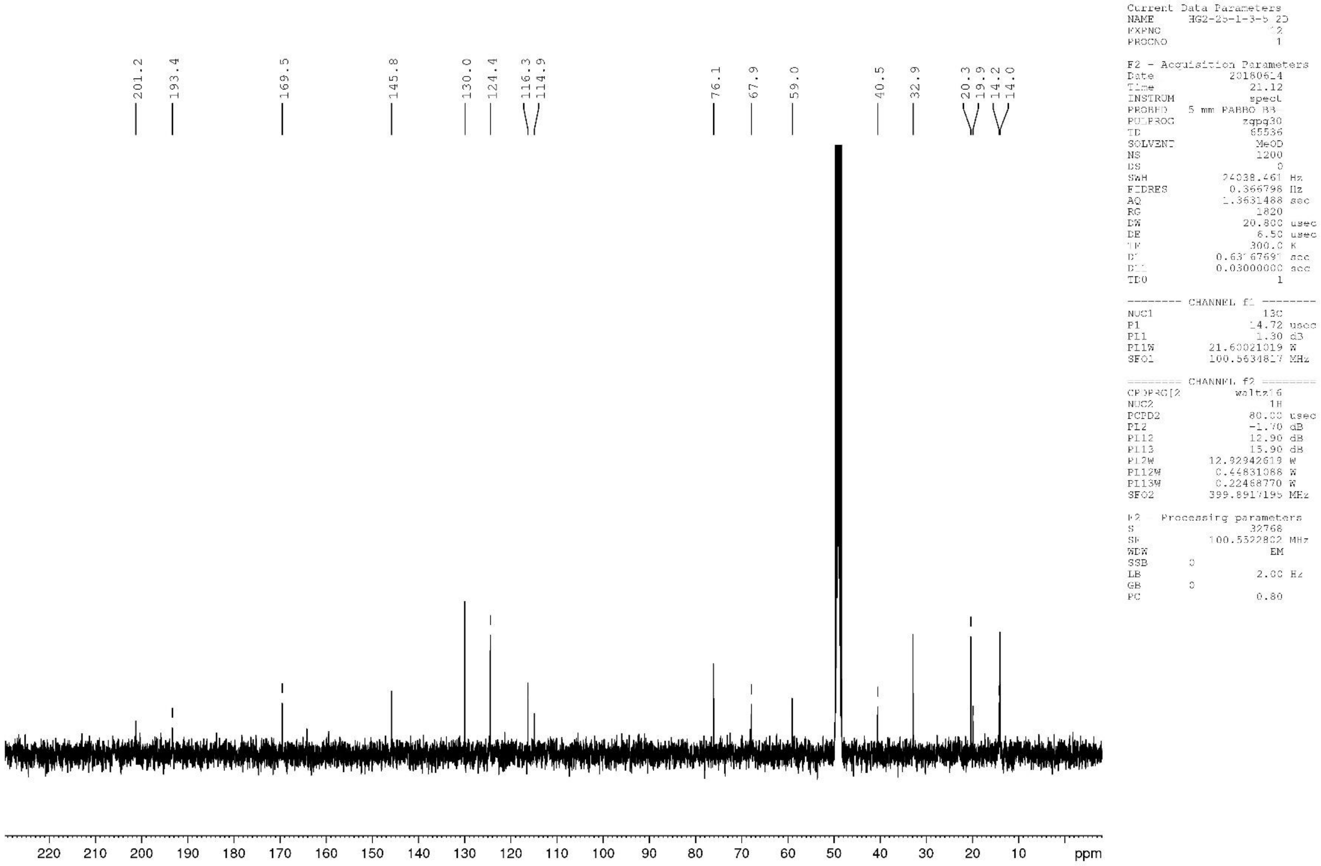

Figure S3 ^{13}C-NMR spectrum (100 MHz, CD_3OD) of a mixture of **1** and **3**

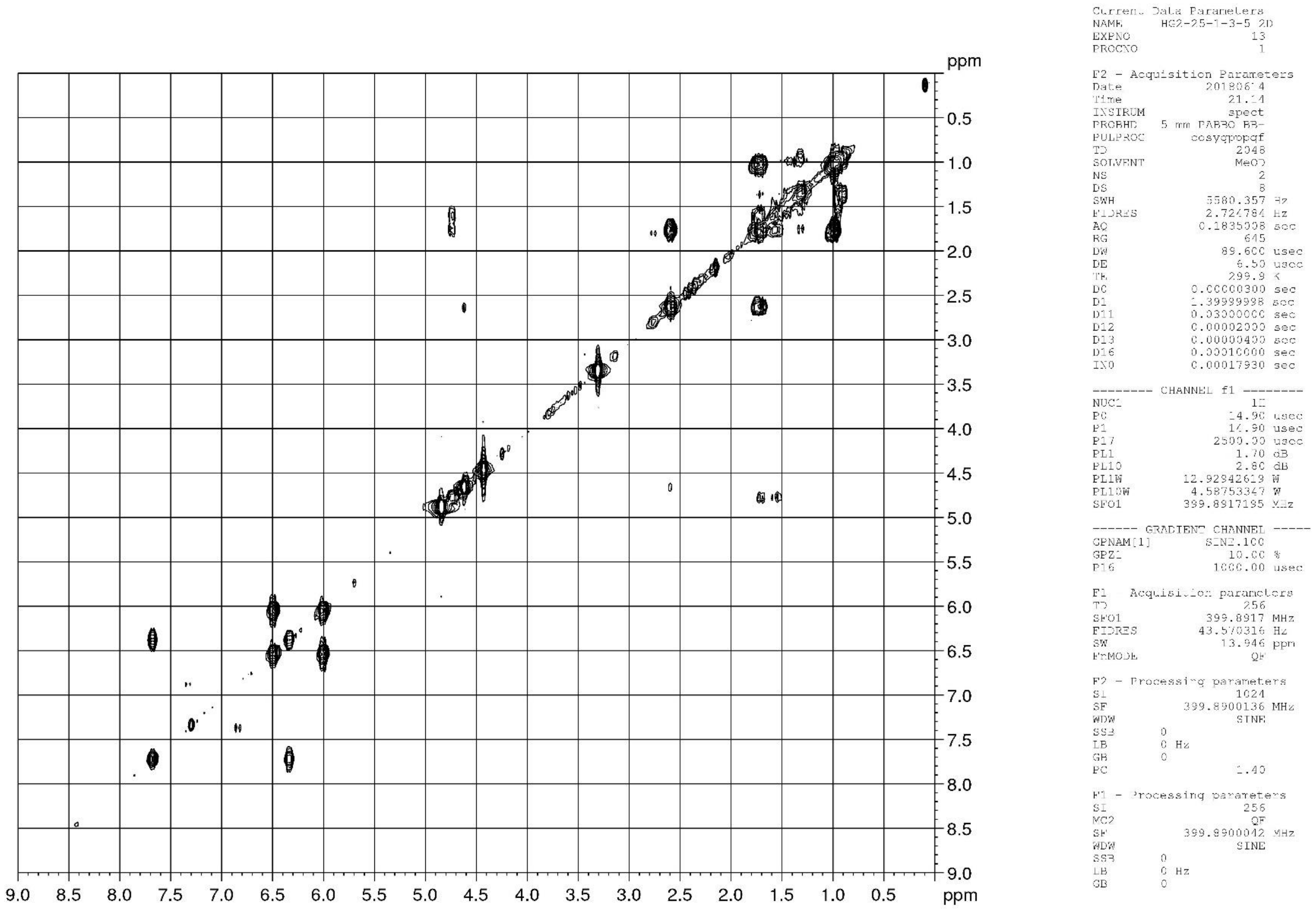

Figure S4 COSY spectrum (CD_3OD) of **1** and **3**

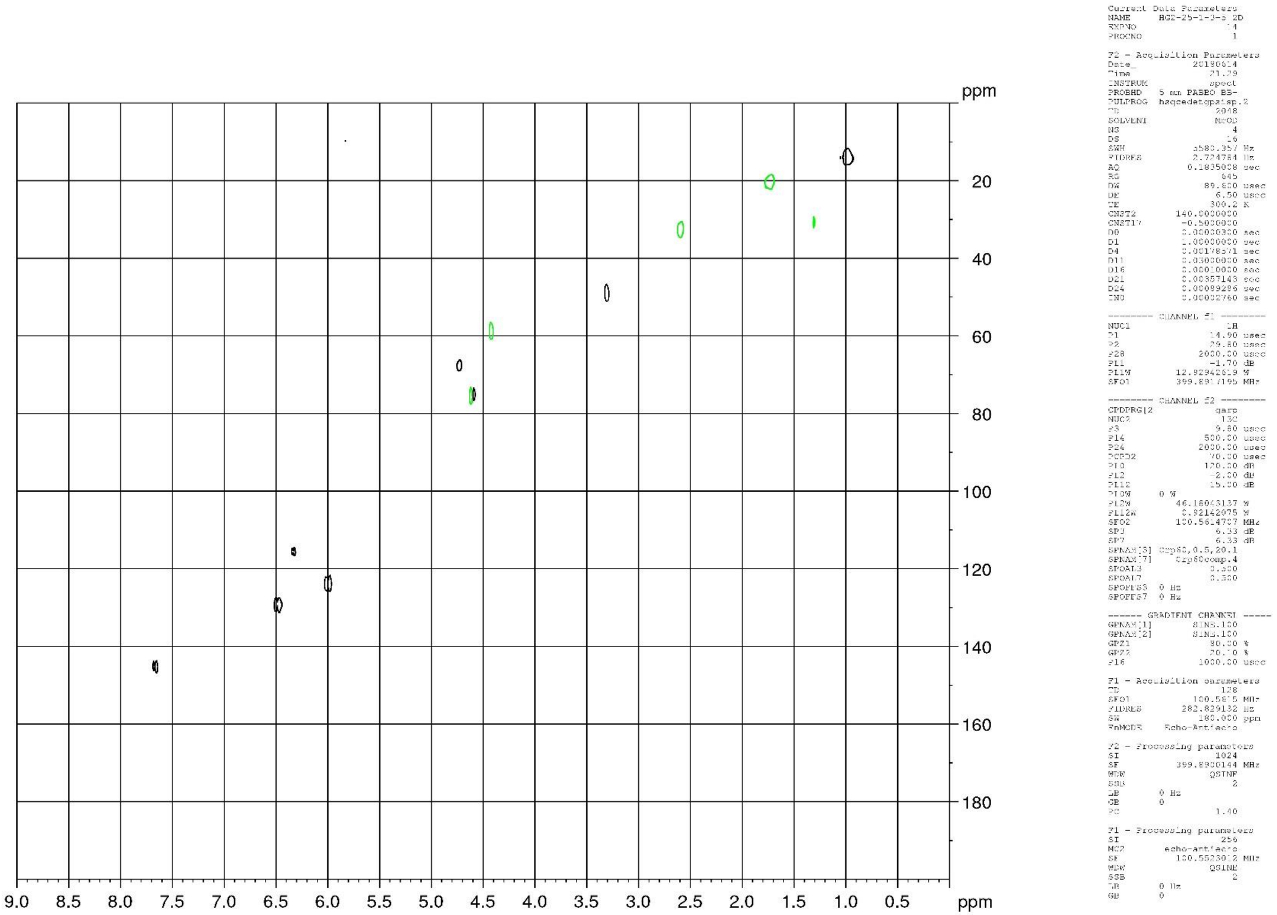

Figure S5 HSQC spectrum (CD_3OD) of **1** and **3**

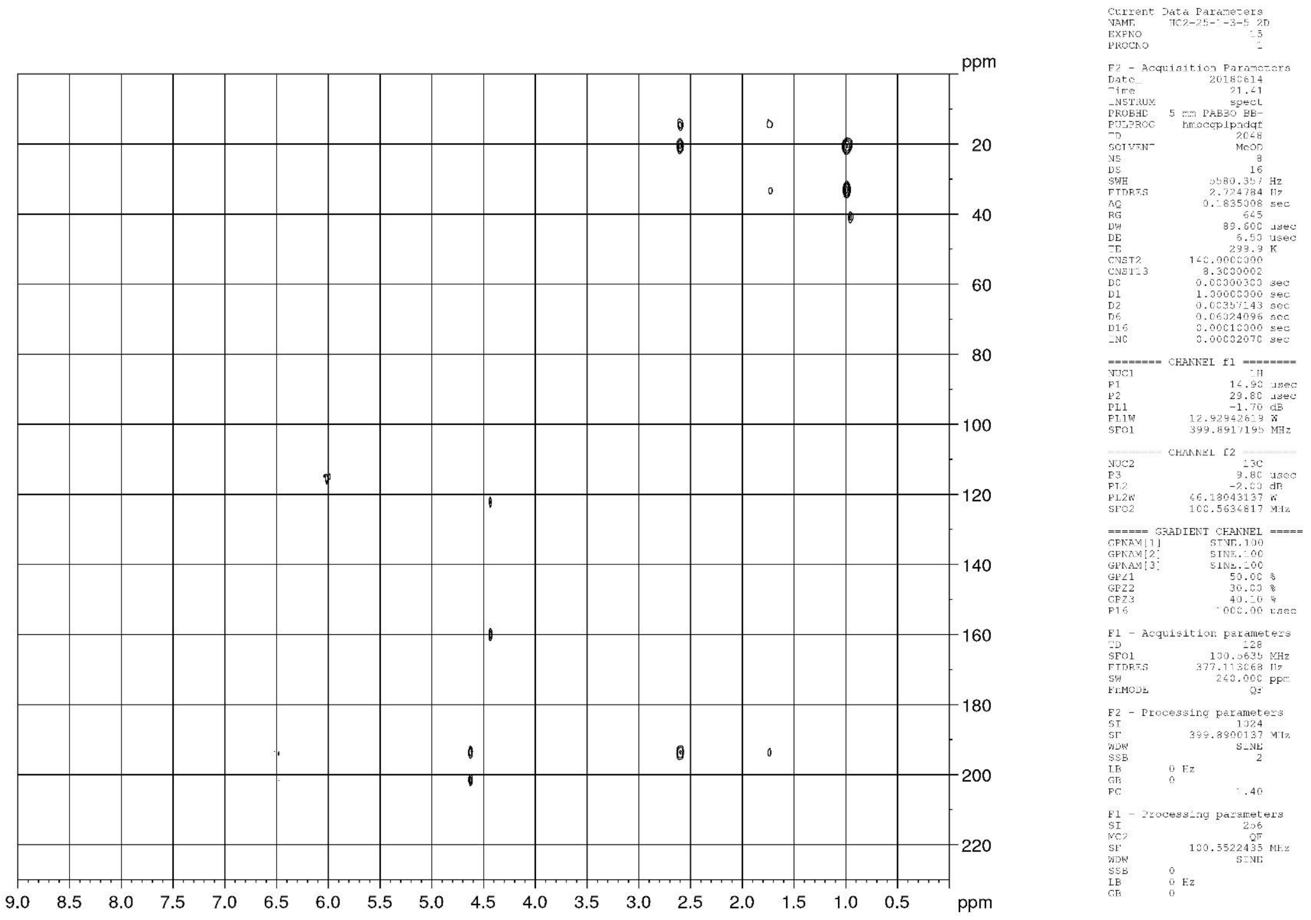

Figure S6 HMBC spectrum (CD_3OD) of **1** and **3**

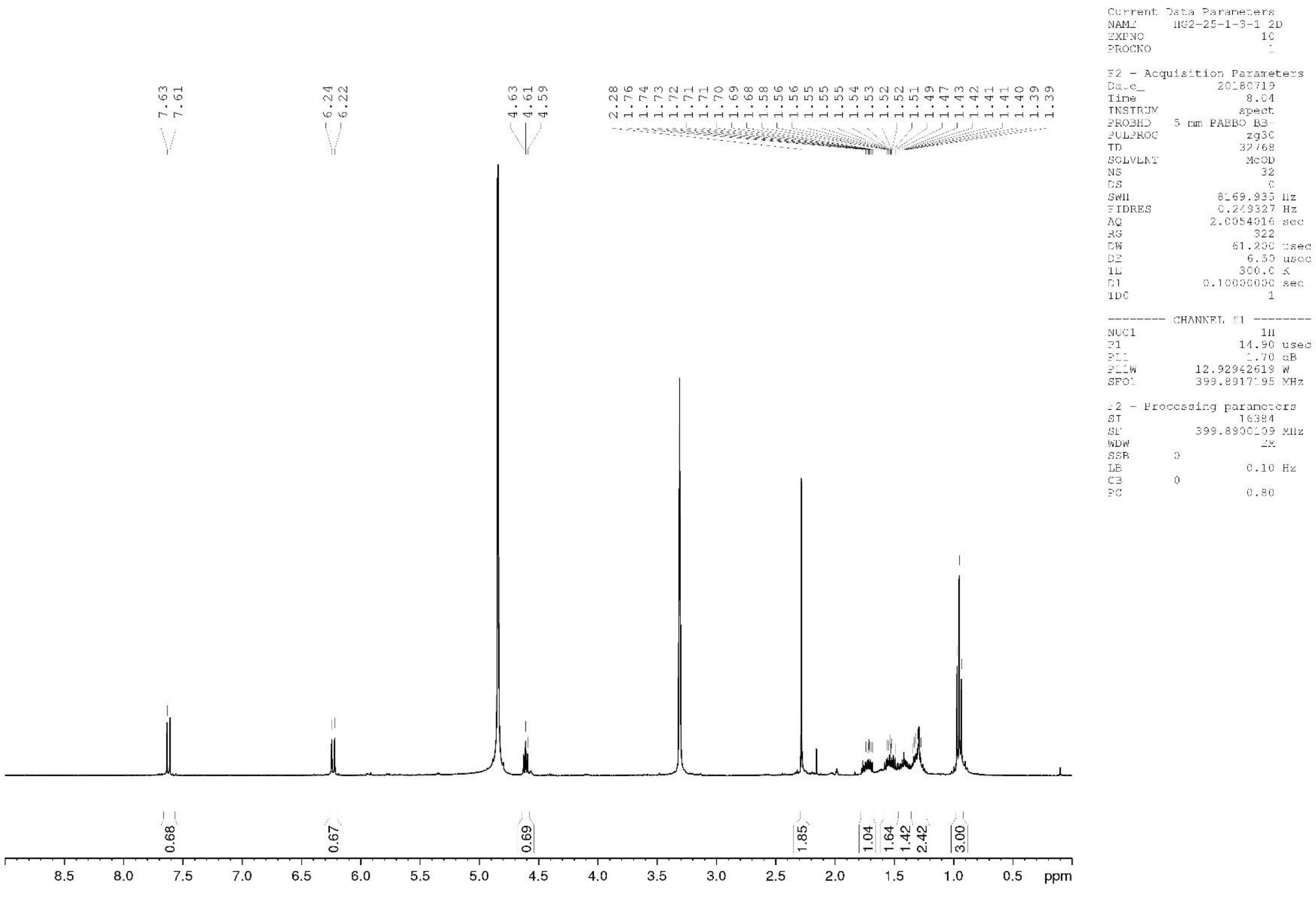

Figure S7 ^{1}H-NMR spectrum (400 MHz, CD_3OD) of **4**

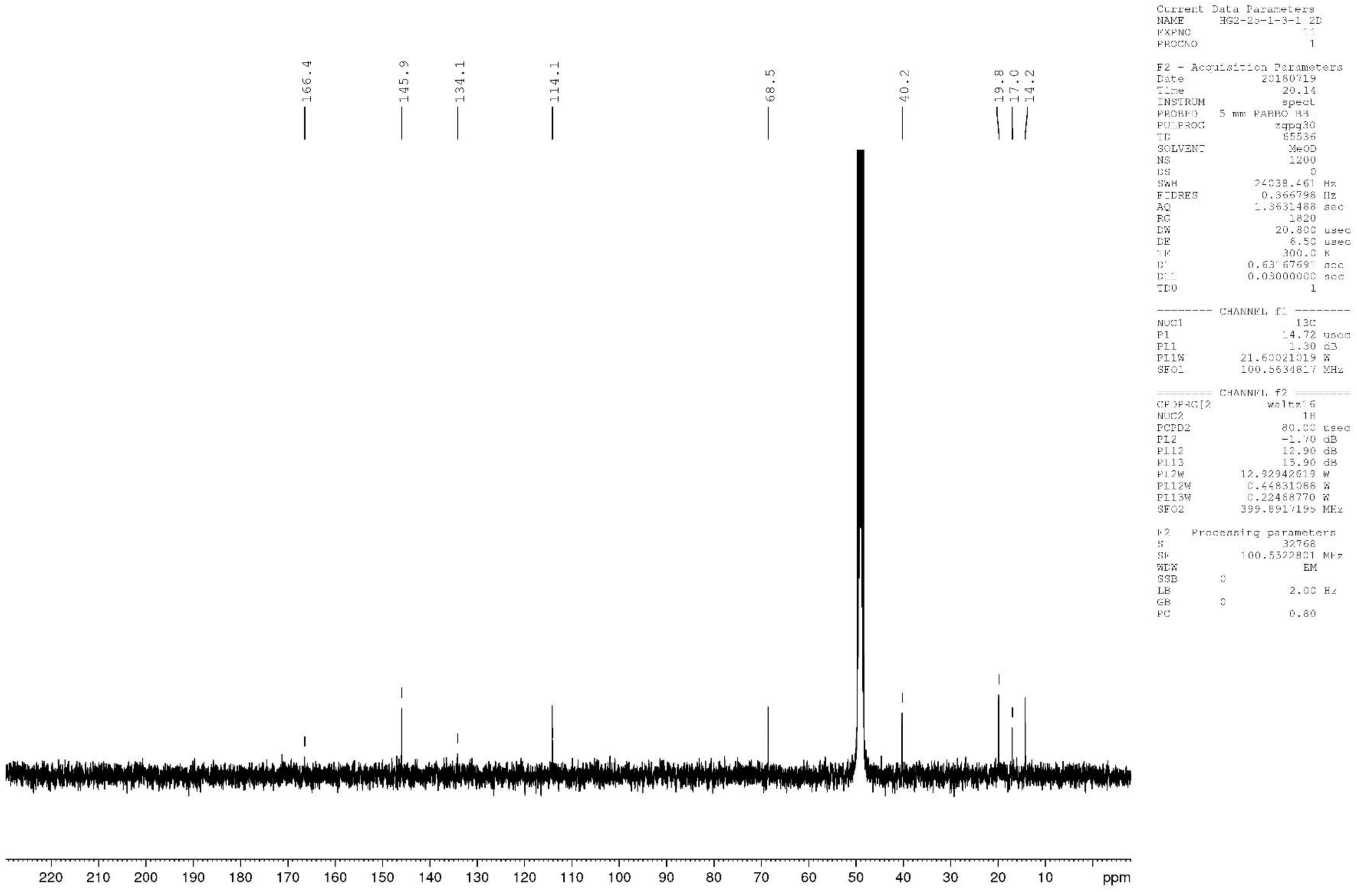

Figure S8 ^{13}C-NMR spectrum (100 MHz, CD_3OD) of **4**

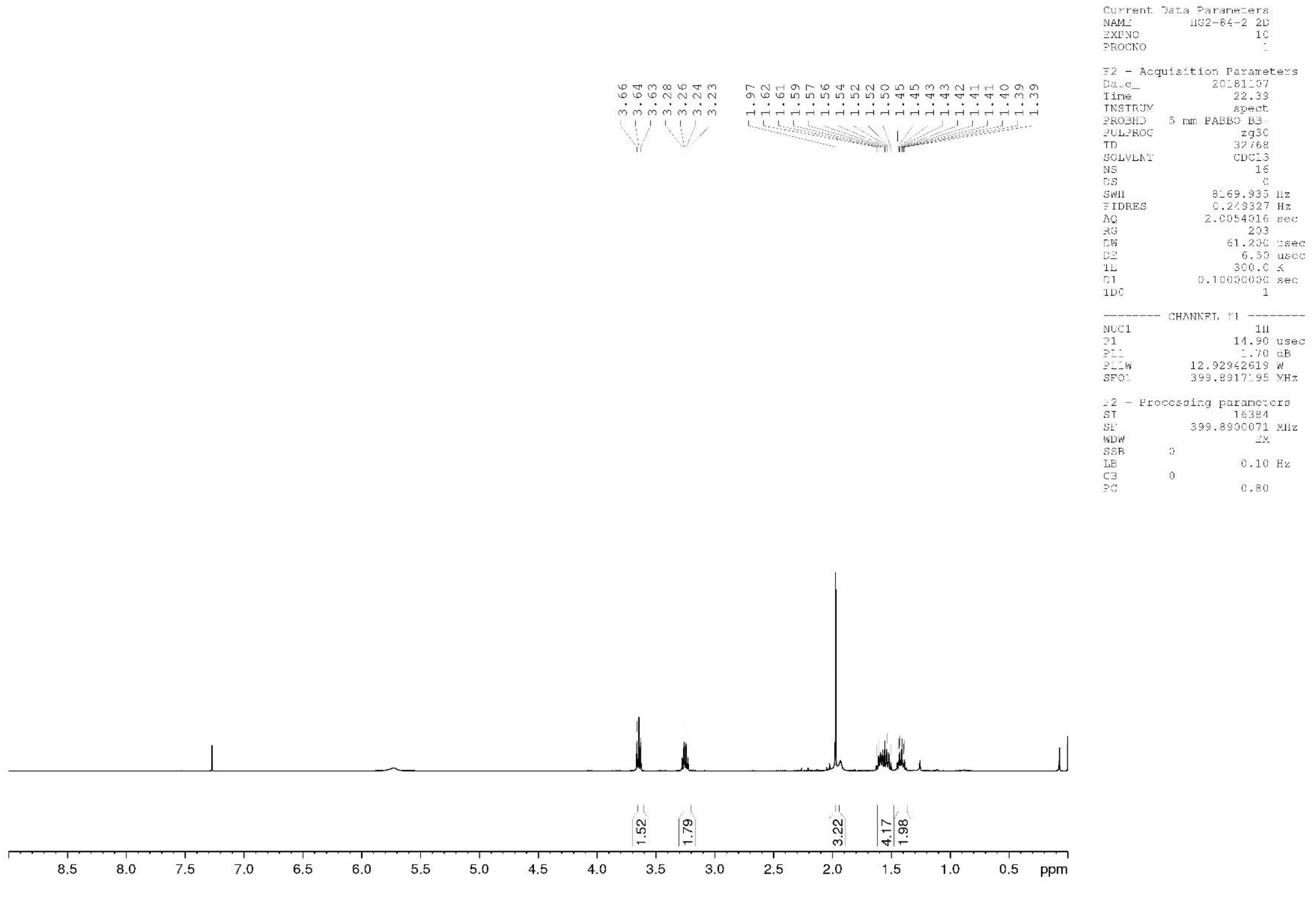

Figure S9 ^{1}H-NMR spectrum (400 MHz, $CDCl_3$) of **6**

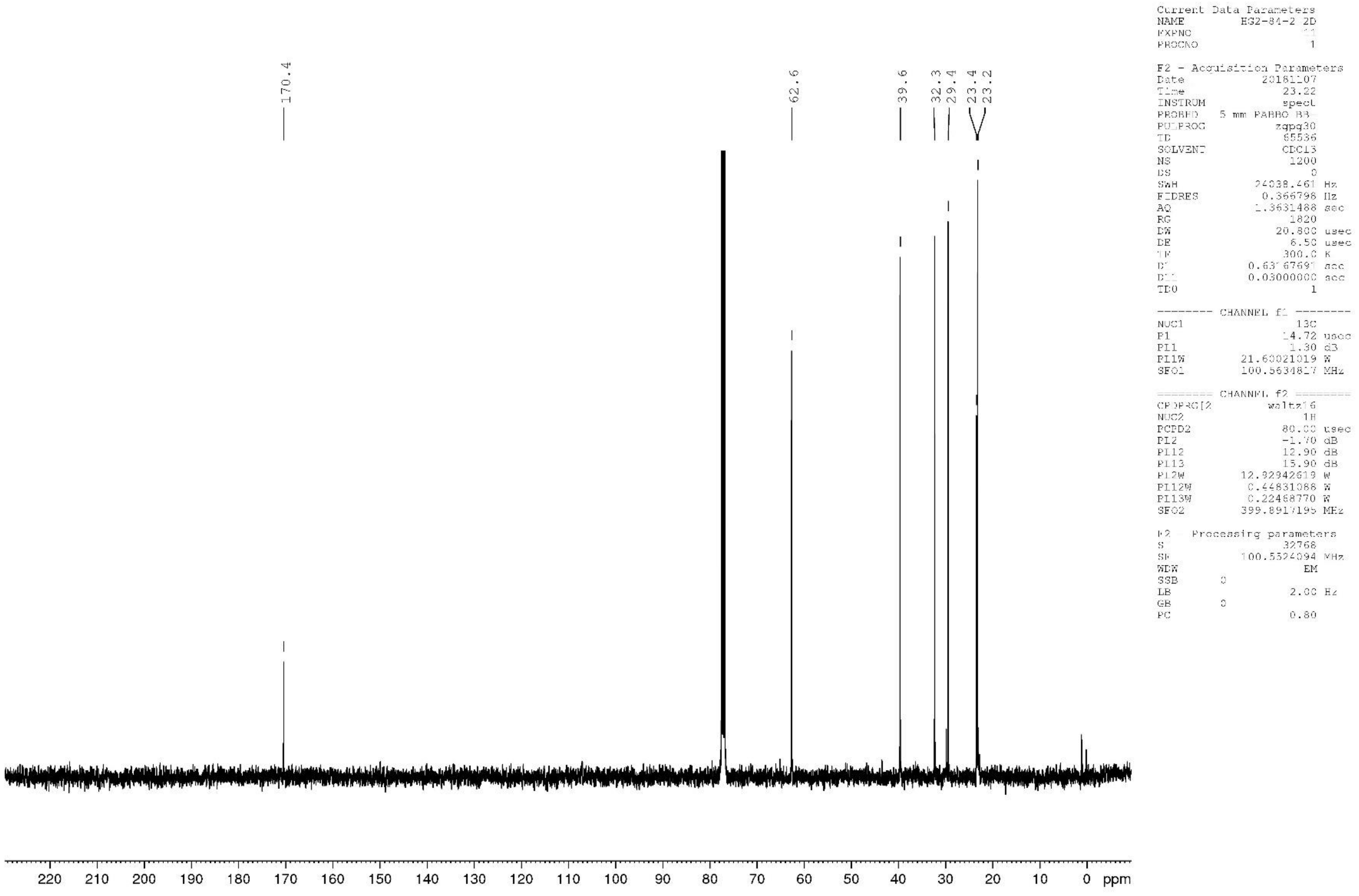

Figure S10 ^{13}C-NMR spectrum (100 MHz, $CDCl_3$) of **6**

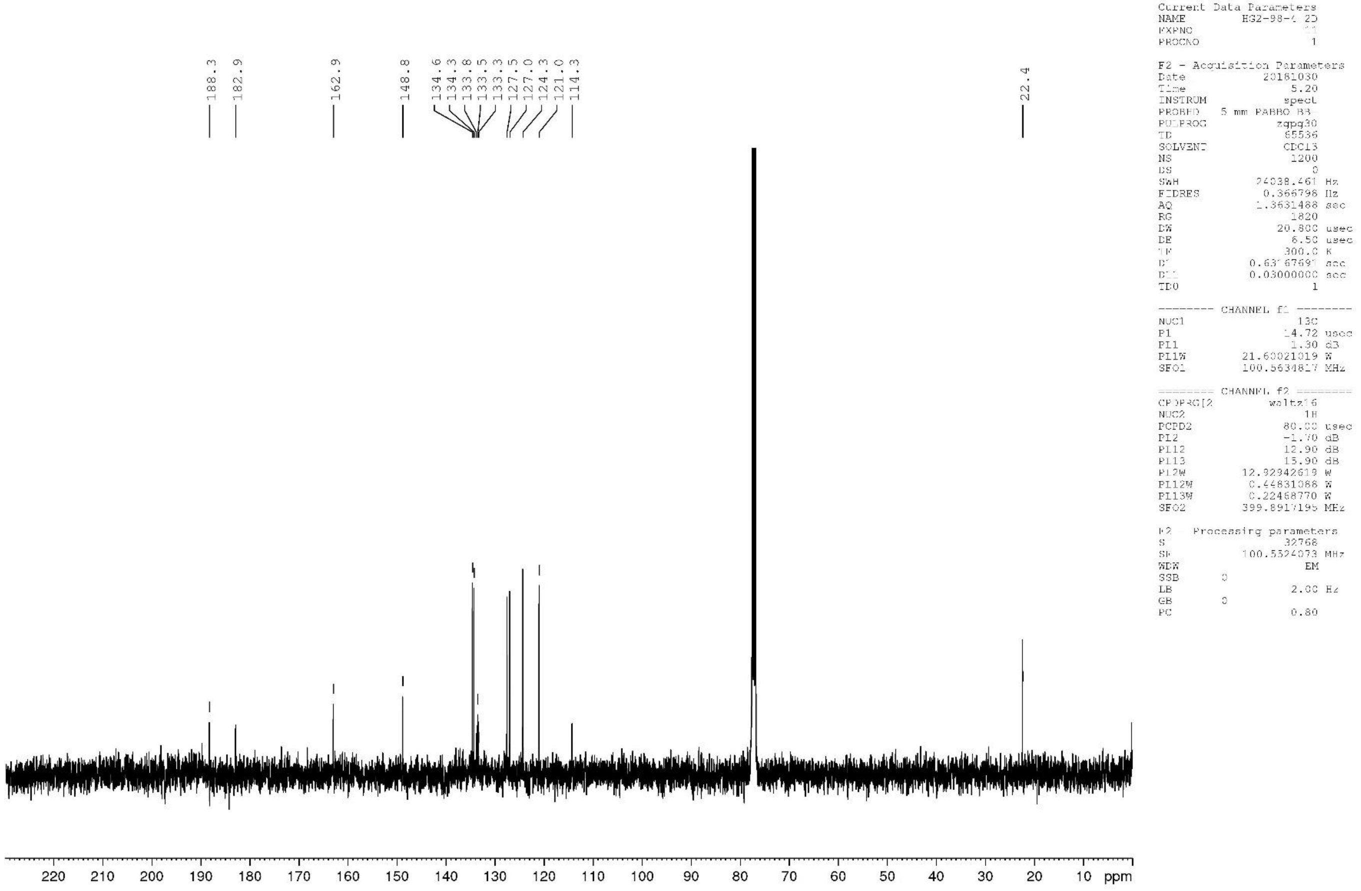

Figure S11 ^{13}C-NMR spectrum (100 MHz, $CDCl_3$) of **5**

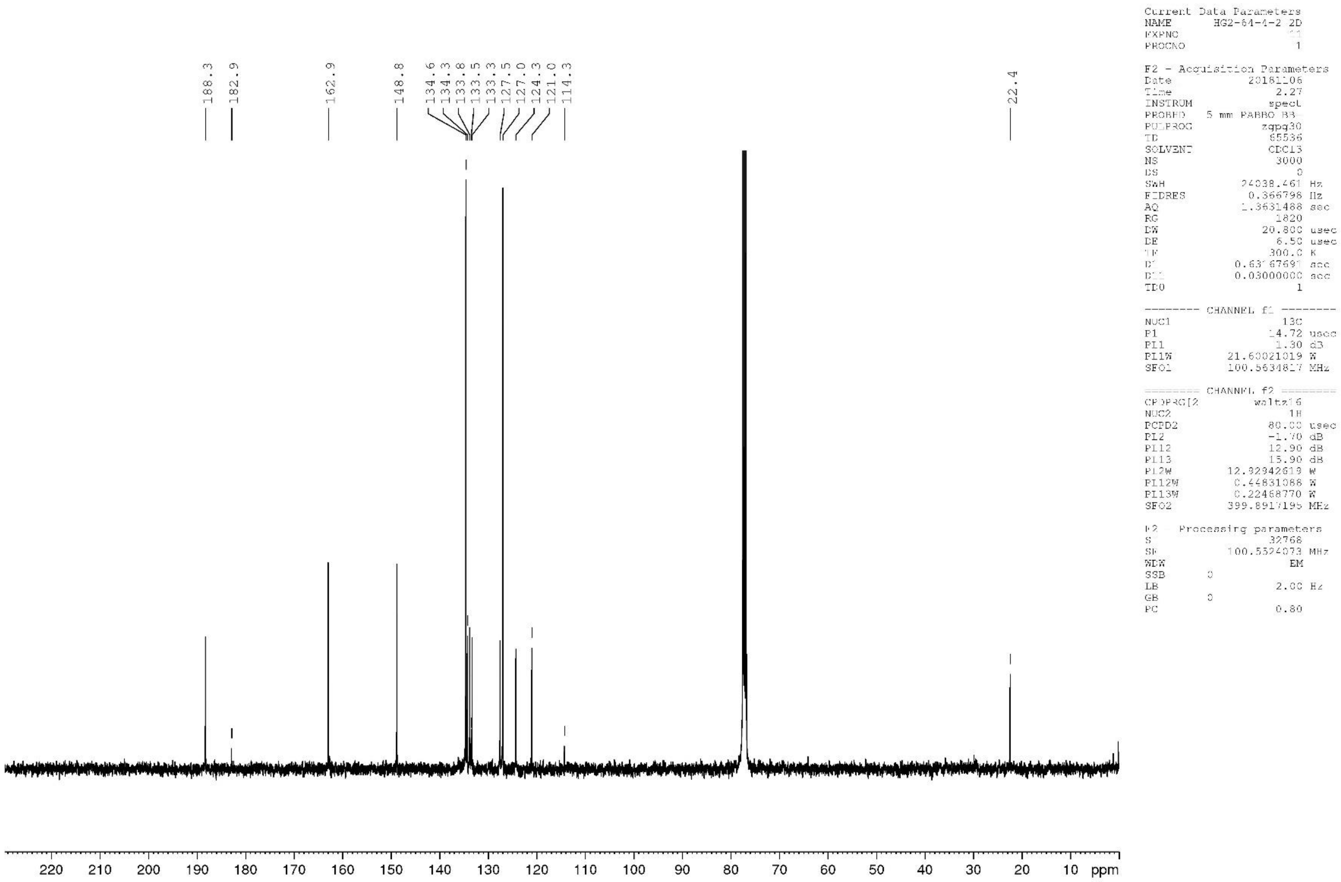

Figure S12 ^{13}C-NMR spectrum (100 MHz, $CDCl_3$) of 1-^{13}C acetate labelled **5**
